# The association and intervention effect between eHealth literacy and lifestyle behaviors among Chinese university students

**DOI:** 10.1590/1980-220X-REEUSP-2022-0147en

**Published:** 2022-12-12

**Authors:** Hua Tian, Jie Chen

**Affiliations:** 1 Xinyang Normal University, College of Life Science, China.; 2 Xinyang Normal University, School of Marxism, China.

**Keywords:** Health Literac, Healthy Lifestyl, Student, China, Letramento em Saúde, Estilo de Vida Saudável, Estudantes, China, Alfabetización en Salud, Estilo de Vida Saludable, Estudiantes, China

## Abstract

**Objective:**

Our aim was to evaluate the association between eHealth literacy and lifestyle behaviors to intervene among Chinese university students.

**Method:**

The Chinese eHealth Literacy Scale (C-eHEALS) questionnaire was used to investigate the eHealth literacy level and association with lifestyle behaviors among Chinese university students. Independent sample t-test and Pearson’s correlation coefficient were used by statistical software SPSS v20.

**Results:**

In the first round, 5,151 university students participated in the study, including 71.46% female and 28.54% male, aged 18–22 (93.13%).The average eHealth literacy score was 26.81 ± 5.83. Four lifestyle behaviors (e.g., exercise, smoking, drinking and sleeping) were all significantly correlated with eHealth literacy scores and demonstrated significant differences. In the second investigation (N = 2,939), the average eHealth literacy score was 31.64 ± 6.44, a notable improvement compared with the first investigation.

**Conclusion:**

Those in the group with high eHealth literacy scores have a healthier lifestyle than those in the low-score group. Training in eHealth-related resources is a positive measure to improve university students’ eHealth literacy.

## RESUMO

**Objetivo:**

Nosso objetivo foi avaliar a associação entre alfabetização em saúde e comportamentos de estilo de vida para intervir entre estudantes universitários chineses.

**Método:**

O questionário Chinese eHealth Literacy Scale (C-eHEALS) foi usado para investigar o nível de alfabetização em saúde e a associação com comportamentos de estilo de vida entre estudantes universitários chineses. O teste t de amostra independente e o coeficiente de correlação de Pearson foram usados pelo *software* estatístico SPSS v20.

**Resultados:**

Na primeira etapa, participaram 5.151 estudantes universitários, sendo 71,46% do sexo feminino e 28,54% do sexo masculino, com idades entre 18 e 22 anos (93,13%). Quatro comportamentos de estilo de vida (exercícios, fumar, beber e dormir) foram todos significativamente correlacionados com os escores de alfabetização em saúde e demonstraram diferenças significativas. Na segunda investigação (N = 2.939), a pontuação média de alfabetização em saúde foi de 31,64 ± 6,44, uma melhoria notável em comparação com a primeira investigação.

**Conclusão:**

Aqueles do grupo com pontuações altas de alfabetização em saúde têm um estilo de vida mais saudável que aqueles do grupo com pontuação baixa. A formação em recursos relacionados com a saúde é uma medida positiva para melhorar a alfabetização em saúde dos estudantes universitários.

## RESUMEN

**Objetivo:**

Evaluar la asociación entre la alfabetización en salud y los comportamientos de estilo de vida para las intervenciones de estudiantes universitarios chinos.

**Método:**

Para investigar el nivel de alfabetización en salud y su asociación con los comportamientos de estilo de vida se utilizó el cuestionario Escala de Alfabetización China en Cibermedicina (C-eHEALS). La prueba t de la muestra independiente y el coeficiente de correlación de Pearson se llevaron a cabo con el software estadístico SPSS v20.

**Resultados:**

En la primera etapa participaron 5.151 estudiantes universitarios, 71,46% mujeres y 28,54% hombres, con edades comprendidas entre los 18 y los 22 años (93,13%). Cuatro conductas de estilo de vida (ejercicio, fumar, beber y dormir) se correlacionaron significativamente con las puntuaciones de Alfabetización en Salud y mostraron diferencias significativas. En la segunda investigación (N = 2.939), la puntuación media de la alfabetización sanitaria fue de 31,64 ± 6,44, lo que supone una notable mejora en comparación con la primera investigación.

**Conclusión:**

Los estudiantes del grupo con puntuación alta de alfabetización en salud tienen un estilo de vida más saludable que las del grupo con puntuación baja. La instrucción sobre recursos relacionados con la salud es una medida positiva para mejorar la alfabetización sanitaria de los estudiantes universitarios.

## INTRODUCTION

With the increasing use of Internet and mobile technology, everyone can access information anywhere and at any time^(1)^. The Internet has become the main source of health-related information^(2)^. More and more teenagers use the Internet to obtain health information^(3,4)^, including university students^(5)^. The ability to find, understand and evaluate health-related information from electronic resources and apply it solving health problems is called eHealth literacy^(6)^. eHealth literacy has been regarded as a public health goal in the 21^st^ century and a major challenge facing global health care. While the current generation of university students can obtain a large amount of health information online, access alone cannot ensure that students skillfully search for such health information; their ability to obtain and evaluate electronic health information still has much room for improvement^(7)^.

University students comprise a special social group, a balanced diet, reasonable nutrition supply and eating habits are particularly important critical to their physical and mental health development. Healthy lifestyle behaviors, such as regular exercise, good sleep, and breakfast, have an important impact on their lives^(8)^. However, university students are prone to engaging in dangerous health behaviors^(9)^, such as smoking and drinking. It is crucial for university students to obtain a healthy lifestyle to protect and promote their health, and to maintain these behaviors in adulthood^(10)^. Lifestyle is defined as voluntary activities of daily life, such activities are a regular part of daily life and can seriously affect one’s health^(11)^. Therefore, eHealth literacy is an important factor for university students in maintaining a healthy lifestyle and good health^(12)^.

The eHealth literacy scale, an 8-item questionnaire designed to measure self-perceived eHealth literacy skills, is comprised of six core skills, or literacies: traditional literacy, health literacy, information literacy, scientific literacy, media literacy, and computer literacy^(6)^. Research showed that in the general population, those with higher levels of eHealth knowledge demonstrated a healthier lifestyle than those with lower levels^(13)^. In the case of college students, few studies have detailed the relationship between eHealth literacy and lifestyle behaviors such as regular exercise, healthy diet and regular sleep, such studies have been conducted in Taiwan, Greece and the United States^(14,15,16)^. The impact of eHealth literacy on lifestyle behavior varied by country, cultural background and Internet use^(2,17,18)^. To date, such studies are lacking in China. Accordingly, we set out to evaluate the eHealth literacy level of Chinese university students and the associations with lifestyle behaviors.

## METHODS

### Study Design

This non-experimental comparative correlation study used quantitative research methods with intervention. The intervention measure carried out more than two eHealth-related trainings for 5,151 participants, and randomly selected 2,939 participants for a second analysis to explore the association between eHealth and lifestyle behaviors among Chinese university students, in order to verify eHealth literacy levels and to measurement the effectiveness of eHealth training intervention.

### Sample Definition

University students were recruited from Xinyang University in China, with the stated goal of examining associations between eHealth literacy and lifestyle behaviors among Chinese university students. Two rounds of investigation were conducted from October to December 2021.

### Selecton Criteria

All participants were native Mandarin speakers majoring in liberal arts and science, from freshman to senior years. Most were aged 18–22 years old. Participants who did not complete the survey were excluded.

### Data Collection Instrument

The research was conducted in two-stages at two months apart. After the first part, participants were trained at least twice in eHealth-related resources. The aim of the second interview was to explore whether eHealth-related training improved eHealth literacy of university students. Nine indicators, including sex, age, only child, origin, parental education level, major, grade, and number of Internet devices were collected, via the Chinese version of the eHealth Literacy Scale (C-EHEALS) questionnaire^(19)^.The C-eHEALS comprises 8 items to which participants respond on a 5–point Likert scale, ranging from 1 for “strongly disagree” to 5 for “strongly agree”, with a total score varying 8 and 40. Data were collected through the online questionnaire created with Wenjuanxing software. Participants were recruited by WeChat, a social media application widely used in China. WeChat was chosen because users are verified, rendering virtually zero possibilities of fake profiles. The researchers sent the questionnaire link to a group of teachers on WeChat and asked them to share it with their students. The questionnaire was anonymous and participants did not receive any payment.

### Data Analysis

Lifestyle behaviors were assessed by the following questions of exercise, breakfast, smoking, drinking and sleeping for statistical analysis. Answers to exercise frequency (none, 1–2 days/month, 1–2 days/week, ≥ 3 days/week) were divided into two groups regular (≥ 1 day/week) and none or unregular (< 1 day/week). Answers to breakfast frequency (none, 1–2 days/week, 3–4 days/week, 5–6 days/week, everyday) were divided into two groups: having breakfast (≥ 1 day/week) and none (< 1 day/week). Answers of smoking (none, 1–2 times/week, 3–4 times/week, everyday) were divided into two groups: smoking (≥ 1 time/week) and none (< 1 day/week). Answers of drinking (none, 1–2 days/month, 1–2 days/week, ≥ 3 times/week) were divided into two groups: drinking (≥ 1 time/month) and none (< 1 day/month). As for sleeping, answers of (≤ 6 hours/day, 7–8 hours/day, ≥ 9 hours/day) were divided into two groups: good (≥ 7 hours/day) and poor (≤ 6 hours/day).

The data are presented through descriptive statistics (mean, standard deviation, frequency, percentage), according to the nature of the variables (quantitative). Reliability and validity of the questionnaire was verified through Kaiser–Meyer–Olkin (KMO) test and Cronbach’s Alpha. The correlation between variables was determined by independent sample t-test and Pearson’s correlation coefficient. The statistical significance was 0.1% and the analyses were assisted by statistical software SPSS v20.

### Ethical Aspects

This study was performed in compliance with the Helsinki Declaration guidelines. All procedures relevant to study participants were approved by the ethics committee of Xinyang University. Participation was voluntary; participants were informed of the study objective and context and provided their written informed consent regarding privacy and information management policies.

## RESULTS

### C-eHEALS Items

The C-eHEALS scores of 5,151 university students ranged from 8 to 40; the mean and standard deviation were 26.81 and 5.83, respectively. The Kaiser–Meyer–Olkin (KMO) test showed an overall KMO value of 0.914. Individual KMO values varied from 0.880 (Item-7) to 0.946 (Item-8). For the eight C-eHEALS items, Cronbach’s Alpha were all above 0.9, varying from 0.921 (Item-3 and Item-4) to 0.929 (Item-7). Thus, the C-eHEALS showed excellent reliability (Total Cronbach’s Alpha = 0.933).

### Associations of Participants Characteristics with C-eHEALS Literacy

The average C-eHEALS score of male was 1.95 higher than that of females (*p* < 0.001, Table 1), with significant difference between the two. The average C-eHEALS score of university students from town was significantly higher than that of countryside (*p* < 0.001), while the C-eHEALS with high parental education was also significantly higher than that of students with low parental education (*p* < 0.001). Overall, the characteristics of sex, region, parental education level, and major had a significant impact on the eHealth literacy scores of Chinese university students.

**Table 1.** Associations of participant characteristics with C-eHEALS literacy score (N = 5,151) – Xinyang, Henan, China, 2021.

Characteristics	C-eHEALS (Mean ± SD)	*P* value
Sex		
Male (1,410)	28.23 ± 6.46	< 0.001
Female (3,741)	26.28 ± 5.48	
Age		
18–22 (4,797)	26.81 ± 5.86	.061
Other (354)	26.94 ± 6.02	
Only Child		
Yes (524)	27.88 ± 6.39	.016
No (4,627)	26.69 ± 5.75	
Origin		
Town (1,135)	27.79 ± 6.27	< 0.001
Countryside (4,016)	26.53 ± 5.66	
Parental education level		
Low (3,953)	26.55 ± 5.68	< 0.001
High (1,198)	27.68 ± 6.20	
Major		
Liberal arts (3,146)	26.60 ± 5.68	= .001
Science (2,005)	27.15 ± 6.04	
Grade		
1 (2,018)	26.82 ± 5.93	.991
> 1 (3,133)	26.81 ± 5.76	
Number of Internet devices		
1(2,042)	26.55 ± 5.72	.004
> 1(3,109)	26.98 ± 5.89	

### Lifestyle Behaviors

For lifestyle behaviors of university students in China, exercise, breakfast, smoking, drinking and sleeping were statistically analyzed (Table 2). To further study the correlation of C-eHEALS with lifestyle behaviors, C-eHEALS was divided into two levels, low (≤ 26) and high (> 26), with an average score of 26 points according to eHealth literacy (Table 2). Comparing lifestyle behaviors, 67.59% of university students in the low group, exercise regularly (≥ 1–2 days/week), compared to 76.75% in the high group. Further, 91.29% vs. 93.76% of participants in the low and high groups regularly eat breakfast, respectively. The proportion of university students who neither smoke nor drink was 96.33% and 90.78% in the low group, respectively, vs. 95.39% and 87.92% in the high group, slightly lower than the former. As for sleeping, 86.05% and 89.28% participants in the low and high C-eHEALS groups slept for above 7–8 hours, respectively. Generally, in the low C-eHEALS group, the participants’ proportion is slightly lower than that of high group, excluding the lifestyles without smoking or drinking. Overall, participants in the high group demonstrated a healthier lifestyle than those in the low group.

**Table 2.** Correlation between C-eHEALS with lifestyle behaviors (N = 5,151) – Xinyang, Henan, China, 2021.

Lifestyle behaviors	Frequency	Sample (%)	C-eHEALSlow (n%)	C-eHEALShigh (n%)	*P* value
Total	5,151.00	100	57.08	42.92	<0.001
Exercise					
None	577.00	11.20	13.13	8.37	<0.001
1–2 days/month	890.00	17.28	19.08	14.88	<0.001
1–2 days/week	2,322.00	45.08	44.66	45.23	<0.001
≥ 3 days/week	1,362.00	26.44	22.93	31.12	<0.001
Breakfast					
None	394.00	7.65	8.71	6.24	<0.001
1–2 days/week	691.00	13.41	14.29	12.26	<0.001
3–4 days/week	944.00	18.33	18.33	18.32	<0.001
5–6 days/week	907.00	17.61	18.20	16.82	<0.001
Everyday	2,215.00	43.00	40.48	46.36	<0.001
Smoking					<0.001
None	4,941.00	95.92	96.33	95.39	<0.001
1–2 times/week	55.00	1.07	1.19	0.90	<0.001
3–4 times/week	42.00	0.82	0.71	0.95	<0.001
Everyday	113.00	2.19	1.77	2.76	<0.001
Drinking					
None	4,613.00	89.56	90.78	87.92	<0.001
1–2 days/month	430.00	8.35	7.38	9.63	<0.001
1–2 days/week	56.00	1.09	0.92	1.31	<0.001
≥ 3 times/week	52.00	1.01	0.92	1.13	<0.001
Sleeping					<0.001
≤ 6 hours/day	647.00	12.56	13.95	10.72	<0.001
7–8 hours/day	4,338.00	84.22	83.27	85.48	<0.001
≥ 9 hours/day	166.00	3.22	2.79	3.80	<0.001

### Associations of Lifestyle Behaviors with C-eHEALS Literacy

To analyze associations of lifestyle behaviors with eHealth literacy, Pearson correlation analysis was conducted (Table 3). As can be seen, there was a significant positive correlation between exercise, smoking, drinking and sleeping with C-eHEALS, respectively. There was no significant correlation between breakfast with C-eHEALS.

**Table 3.** Pearson correlation analysis (N = 5,151) – Xinyang, Henan, China, 2021.

Characteristics	C-eHEALS	Exercise	Breakfast	Smoking	Drinking	Sleeping
C-eHEALS	1					
Exercise	0.119**	1				
Breakfast	0.021	0.071**	1			
Smoking	0.032*	0.030*	0.021	1		
Drinking	0.041**	0.046**	–0.011	0.396**	1	
Sleeping	0.054**	0.080**	0.007	–0.062**	–0.061**	1

Note. ** indicates a significant correlation at the 0.01 level (bilateral). * indicates a significant correlation at the 0. 05 level (bilateral).

To validate the eHealth literacy level of university students and to measure the effectiveness of eHealth training intervention, a second round of questionnaire survey was launched two months later (Table 4). A total of 2,939 university students who had participated the first survey were randomly selected to carry out the second survey. Between surveys and the respondents were trained twice or more on eHealth literacy. Results show that participants in the high group have a healthier lifestyle than those in the low group, consistent with the first survey. The mean and standard deviation of eHealth literacy increased from 26.81 ± 5.83 (N = 5,151) to 31.64 ± 6.44 (N = 2,939). Compared with the first survey, the C-eHEALS score of five lifestyle behaviors improved, while with regular lifestyle had a higher C-eHEALS score than those with irregular lifestyle (Table 5). Based on unhealthy lifestyle behaviors of the two-stage study, such as five lifestyle behaviors of exercise (none), breakfast (none), drinking (≥ 3 times/week), sleeping (≤ 6 hours/day) and smoking (everyday), the participants in both steps had a low C-eHEALS score. Thus, eHealth training is a positive intervention.

**Table 4.** Verifying the first results (N = 2,939) – Xinyang, Henan, China, 2021.

Lifestyle behaviors	Frequency	Sample (%)	C-eHEALSlow (n%)	C-eHEALShigh (n%)	*P* value
Total	2,939.00	100	44.27	55.73	<0.001
Exercise					
None	217.00	7.38	9.61	5.62	<0.001
1–2 days/month	323.00	10.99	12.91	9.46	<0.001
1–2 days/week	1,341.00	45.63	49.04	42.92	<0.001
≥ 3 days/week	1,058.00	36.00	28.44	42.00	<0.001
Breakfast					
None	228.00	7.76	9.15	6.65	<0.001
1–2 days/week	353.00	12.01	12.61	11.54	<0.001
3–4 days/week	548.00	18.65	19.29	18.13	<0.001
5–6 days/week	589.00	20.04	23.29	17.46	<0.001
Everyday	1,221.00	41.54	35.66	46.21	<0.001
Smoking					<0.001
None	2,795.00	95.10	94.77	95.36	<0.001
1–2 times/week	44.00	1.50	1.61	1.40	<0.001
3–4 times/week	23.00	0.78	0.85	0.73	<0.001
Everyday	77.00	2.62	2.77	2.50	<0.001
Drinking					
None	2,606.00	88.67	88.85	88.52	<0.001
1–2 days/month	263.00	8.95	8.99	8.91	<0.001
1–2days/week	31.00	1.05	1.08	1.04	<0.001
≥ 3 times/week	39.00	1.33	1.08	1.53	<0.001
Sleeping					<0.001
≤ 6 hours/day	377.00	12.83	13.84	12.03	<0.001
7–8 hours/day	2,462.00	83.77	84.01	83.58	<0.001
≥ 9 hours/day	100.00	3.40	2.15	4.40	<0.001

**Table 5.** Comparison with two studies (N = 5,151 and N = 2,939) – Xinyang, Henan, China, 2021.

Characteristics	C-eHEALS		P value
	Study 1 (N = 5,151)	Study 2 (N = 2,939)	
Total	26.81±5.83	31.65 ± 6.44	<0.001
Exercise			
None	25.10±5.88	29.79 ± 6.44	<0.001
1–2 days/month	26.06±5.16	30.49 ± 6.40	<0.001
1–2 days/week	26.87±5.55	31.21 ± 6.41	<0.001
≥ 3 days/week	27.92 ± 6.40	32.93 ± 6.52	<0.001
Breakfast			
None	25.96 ± 5.59	30.76 ± 6.44	<0.001
1–2 days/week	26.32 ± 5.53	31.01 ± 6.45	<0.001
3–4 days/week	26.77 ± 5.60	31.31 ± 6.45	<0.001
5–6 days/week	26.44 ± 5.59	30.43 ± 6.57	<0.001
Everyday	27.28 ± 6.09	32.74 ± 6.56	<0.001
Smoking			<0.001
None	26.76 ± 5.75	31.68 ± 6.44	<0.001
1–2 times/week	26.89 ± 6.48	32.16 ± 7.11	<0.001
3–4 times/week	28.52 ± 6.54	29.61 ± 7.49	<0.001
Everyday	28.34 ± 7.79	30.68 ± 7.51	<0.001
Drinking			
None	26.71 ± 5.76	31.62 ± 6.44	<0.001
1–2 days/month	27.63 ± 5.79	31.89 ± 6.48	<0.001
1–2 days/week	27.71 ± 5.80	31.32 ± 7.68	<0.001
≥ 3 times/week	27.69 ± 5.83	32.08 ± 8.81	<0.001
Sleeping			<0.001
≤ 6 hours/day	26.10 ± 6.18	31.06 ± 7.07	<0.001
7–8 hours/day	26.87 ± 5.76	31.64 ± 6.41	<0.001
≥ 9 hours/day	28.10 ± 5.83	34.06 ± 6.44	<0.001

Overall, those in the high group demonstrate a healthier lifestyle than those in the low group. After eHealth literacy training, the ability to identify and evaluate eHealth information had improved. Training in eHealth related resources is an effective way of improving the eHealth literacy of university students.

## DISCUSSION

Herein, the first average score of C-eHEALS was 26.81, lower than that of young adults in Pakistan (29)^(20)^, American university students (31.9)^(16)^, Jordanian nursing students (28.96)^(21)^, or adults in Iran (28.2)^(18)^ and Kuwait (28.6)^(22)^. The score was also lower than that of nursing university students in Sri Lanka (28.02)^(23)^ and Japan medical departments (27.0)^(12)^, and firstyear students in the Faculty of Health Sciences at a university in Turkey (27.5)^(4)^. In certain Asian, American and European countries, the eHealth scores of the general population were also relatively high, ranging from 28.1 to 30.5^(24,25)^. Although the score in this study is low, it is still higher than that of Japanese adults (23.4)^(26)^ and university students(23.6)^(23)^. The eHealth literacy score increased to 31.64 after participants trained for twice or more, indicating that the training of eHealth-related resources is effective in improving university students’ eHealth literacy.

During the COVID-19 pandemic, the Internet has been a particularly important mode for the dissemination of information. China offers a higher and more reliable source of health information, providing people with knowledge and suggestions on COVID-19 health protection than before^(27)^. The Chinese government has increasingly aimed to provide health-related information on social media, while numerous American and international websites provides health and medical information with Chinese^(28)^. Thus, eHealth literacy training has the potential to quickly improve the eHealth literacy of university students. Conversely, the relative lack of an effective online medical system in Japan is considered central to the low level of eHealth literacy in Japan compared with Europe^(29)^. Accordingly, colleges and universities in China and elsewhere should strengthen health and medical science education, optimize network health information services, fortify the training of eHealth-related resources, and provide a good environment for improving the evaluation and application ability of college students’ eHealth information. These measures will help discourage so as to urge them to change dangerous lifestyle behaviors and improve overall quality of life.

Among limitations of this study only eHealth-related resource training intervention was chosen without other intervention measures for comparison. Health professionals require further eHealth literacy research to explore a variety of interventions, in order to help university students achieve healthier lifestyle behaviors.

## CONCLUSIONS

This study used quantitative research methods with intervention to comprehensively analyze the intervention and association between eHealth literacy and lifestyle behaviors among Chinese university students. We found a positive association between university students’ eHealth literacy and lifestyle behaviors. Those in the high-score eHealth literacy group demonstrate a healthier lifestyle than those in the low-score group. Training in health-related resources was a useful measure in improving university students’ eHealth literacy. Most college students have poor exercise behaviors, a factor that requires special consideration when carrying out eHealth-related resource training, especially for the low eHealth literacy group.
